# *Arachis hypogaea* L. Root Extract Mitigates Testosterone Propionate‐Induced Benign Prostatic Hyperplasia in ICR Mice by Suppressing Inflammation, Androgen Receptors, and Dihydrotestosterone

**DOI:** 10.1002/fsn3.71682

**Published:** 2026-03-23

**Authors:** Samee‐Ullah, Faryal Shaukat, Muhammad Umair Ijaz, Muhammad Bilal, Anees Ahmed Khalil, Tausif Ahmad, Yemin Guo, Xia Sun, Yuanda Song, Mohammed Mansour Quradha

**Affiliations:** ^1^ School of Agricultural Engineering and Food Science Shandong University of Technology Zibo P.R. China; ^2^ Shandong Provincial Engineering Research Center of Vegetable Safety and Quality Traceability Zibo Shandong P.R. China; ^3^ University Institute of Diet and Nutritional Sciences The University of Lahore Lahore Pakistan; ^4^ Food Chemistry and Nutrition Science Laboratory, College of Agriculture Virginia State University Petersburg Virginia USA; ^5^ Shandong Engineering Research Center of Precision Nutrition and Healthy Aging Zibo P.R. China; ^6^ Research Institute of Natural Products and Health Industry Innovation Qilu Medical University Zibo City P.R. China; ^7^ College of Education Seiyun University Seiyun Hadhramawt Yemen; ^8^ Pharmacy Department, Medical Sciences Aljanad University for Science and Technology Taiz Yemen

**Keywords:** AHRE, alternate therapy, BPH, sex hormone

## Abstract

In traditional Chinese medicine, *Arachis hypogaea* L. root extract (AHRE), and other parts of the plant historically have been used to manage benign prostatic hyperplasia (BPH). Hence, to evaluate the therapeutic effect of AHRE on testosterone‐induced BPH in ICR mice, BPH was induced by daily subcutaneous testosterone propionate injections (6 mg/kg BW) in olive oil for 30 days. AHRE was orally administered at 100, 200, and 300 mg/kg BW daily, with finasteride (1 mg/kg BW) used as the positive control. HPLC‐qTOF‐MS/MS identified 19 compounds in 70% ethanolic AHRE, with resveratrol quantified at 1.12 mg/g dry weight. Prostate weight (PW), prostatic index (PI), histopathological changes, serum concentrations of prostatic acid phosphatase (PAP), dihydrotestosterone (DHT), testosterone (T), estradiol (E_2_), and T/E₂ ratio, along with androgen receptor (AR) levels and the relative mRNA expression of *hypoxia‐inducible factor‐1α* (*HIF‐1α*), *estrogen receptors* (*ER‐α and ER‐β*), *lipoxygenase‐5* (*LOX‐5*), and *cyclooxygenase‐2* (*COX‐2*) were measured. High‐dose AHRE (300 mg/kg) reduced PW by 84.07%, significantly reduced glandular hyperplasia, prostatic cell counts, PAP, DHT, and AR levels (*p* < 0.05). It also downregulated relative mRNA expression of inflammation‐related genes, including *ER‐α* (1.6‐fold), *5‐LOX* (1.65‐fold), and *COX‐2* (1.36‐fold). Preliminary gut microbiota analysis revealed treatment‐associated increases in the relative abundance of *Bifidobacterium*, which showed robust negative correlations with PW (*ρ* = −0.95, *q* < 0.001), PI (*ρ* = −0.96, *q* < 0.001), and inflammatory markers, a genus linked to enhanced gut barrier function and control of systemic inflammation, potentially contributing to the observed improvements in BPH phenotypes.

## Graphical abstract

In summary this study evaluated the protective effects of *Arachis hypogaea* L. root extract (AHRE) against testosterone propionate (TP)‐induced benign prostatic hyperplasia (BPH) in ICR mice. AHRE was prepared and administered in varying doses (100, 200, 300 mg/kg BW) alongside TP, with finasteride as a positive control. Biochemical, histological, and gene expression analyses revealed that AHRE reduced key BPH markers such as DHT, AR, COX‐2, and LOX‐5, while modulating estrogen receptor expression. Additionally, exploratory AHRE study on mice gut microbiota underscores the presence of a dynamic gut–prostate axis in this BPH model and identifies candidate microbial targets—*Bifidobacterium*, *Akkermansia*, *Ruminococcus*, and *Alistipes*.

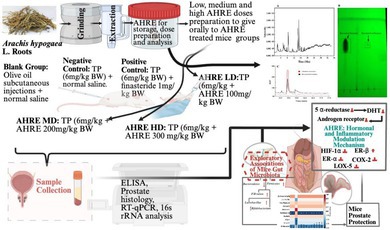

## 1 Introduction

Diseases in the aging population are the main concern in the modern era. Elderly persons are prone to several disorders, including benign prostatic hyperplasia (BPH). The onset of BPH can occur as early as 40 years of age; it progresses slowly, and by 80 years of age, over 90% of men have developed BPH (Wei et al. 2025). Clinical symptoms of BPH are typically classified into lower urinary tract symptoms (LUTS), including storage symptoms (e.g., hesitancy, nocturia, frequency, urgency) and obstructive symptoms (e.g., weak stream), as well as obstructive uropathy (Launer et al. 2021; Wei et al. 2025). Although the exact etiology and pathophysiology of BPH remain unclear, an imbalance in androgenic hormones is believed to contribute to its development (Vickman et al. 2020). Moreover, clinical investigations have demonstrated that chronic inflammation in the prostate tissue contributes to BPH (Launer et al. 2021) and increases the risk of prostate cancer (Chen et al. 2024). Detecting prostate cancer becomes challenging in patients with BPH, as an enlarged prostate can obscure small tumors during digital rectal examination. Additionally, the aggressive nature of prostate cancer cells often leads to treatment resistance, ultimately resulting in castration‐resistant prostate cancer (CRPC) (Mirzaei et al. 2022). As prostate cancer progresses, it can cause pain, urinary difficulties, and other symptoms that significantly impact quality of life (QoL). Despite advancements in treatment, prostate cancer remains a leading cause of cancer‐related deaths in men, highlighting the critical need for ongoing research and the development of more effective therapies.

In terms of therapeutic efficacy, surgical interventions for BPH demonstrate greater reliability compared to pharmacological treatments; however, they are associated with an increased risk of secondary complications. The FDA‐approved medications dutasteride and finasteride, classified as 5*α*‐reductase inhibitors (5ARIs), are widely recognized for their effectiveness in reducing prostate volume by suppressing the dihydrotestosterone (DHT) levels (Dahm et al. 2017). Despite their efficacy, prolonged use of these agents is linked to a range of adverse effects, including a potential increased risk of prostate cancer (Smith and Carson 2009).

The utilization of *Arachis hypogaea* L. plant components in traditional Chinese medicine for inflammatory diseases originated in Ming Hongwu period (1368–1398) and historical texts, including the *Yunnan Great Dictionary of Chinese Medicine* and *Traditional Chinese Medicine Classics*, have documented its therapeutic application (Zu et al. 2014). Although its use has been documented (Bei et al. 2018), contemporary scientific studies investigating the effective dose and underlying mechanisms in testosterone propionate (TP)‐induced BPH models remain limited. Hence, the need to evaluate the preliminary mechanisms underlying its effectiveness prompted this study. Moreover, to explore potential indirect mechanisms underlying AHRE's effects, we performed an exploratory gut microbiota analysis. This revealed associations between AHRE treatment and increased abundance of beneficial taxa, aligning with emerging evidence on the gut‐prostate axis.

## 2 Materials and Methods

### 2.1 Materials and Reagents

Roots of *Arachis hypogaea* L. (peanut) were sourced from a local supplier in Shandong Province, China (batch no. ZBPR202111‐3). A voucher specimen was deposited in the herbarium of the School of Agricultural Engineering and Food Science, Shandong University of Technology, Zibo, Shandong, China. All other reagents and solvents used in this study were of analytical grade or higher. TP and finasteride were purchased from Merck (Darmstadt, Germany). Formalin was purchased from Sigma‐Aldrich (Steinheim, Germany). Serum levels of DHT, testosterone (T), estradiol (E_2_), prostatic acid phosphatase (PAP), and androgen receptor (AR) were measured using commercially available ELISA kits according to the manufacturers' instructions. DHT was quantified using an ELISA kit from Invitrogen (Thermo Fisher Scientific; cat. no. EEL221), T using an ELISA kit from Abcam (cat. no. ab285350), E_2_ using an ELISA kit from Abbexa (cat. no. abx515682), PAP using an ELISA kit from Assay Genie (cat. no. MOFI00764), and AR using an ELISA kit from Biomatik (cat. no. EKU02343). The TIANamp Stool DNA kit (DP328) was purchased from TIANGEN Biotech Co. Ltd. (China).

### 2.2 Extract Standardization

The roots of *A. hypogaea* L. (2 kg) were thoroughly washed, shade‐dried, pulverized into a fine powder, and subjected to three extraction cycles using 70% ethanol (v/v) at 80°C for 3 h each, employing reflux extractors using the previously reported method of Wang et al. 2024 with minor modification (Wang et al. 2024). The extraction procedure yielded a crude extract with consistent batch‐to‐batch recovery of 8.5%–9.3% (w/w). The prepared AHRE was qualitatively analyzed using an Agilent 1100 Liquid Chromatography (LC) system (Agilent Technologies, Santa Clara, CA, USA) interfaced with a quadrupole time‐of‐flight mass spectrometer (qTOF‐MS) via electrospray ionization (ESI). The system was equipped with a standard autosampler. Chromatographic separation was performed on a Phenomenex Gemini C18 column (e.g., 150 mm × 2.0 mm i.d., 5 μm particle size, 110 Å pore size; Phenomenex, Torrance, CA, USA) maintained at 40°C. The mobile phase consisted of 0.1% formic acid in water (A) and 0.1% formic acid in acetonitrile (B) at a flow rate of 0.5 mL/min, using a gradient elution program adapted from previously reported methods (e.g., 5%–95% B over 30 min) with minor modifications (Ullah et al. 2019; Wang et al. 2024). Additionally, resveratrol content in AHRE was quantified following fractionation via silica gel column chromatography, using a solvent system of ethyl acetate:methanol:water with increasing polarity ratios (10:1:1, 7:2:1, and 5:3:2). Furthermore, the quantification was performed using RP‐HPLC on an Agilent 1100 Series system with a ZORBAX Eclipse Plus C18 column (150 mm × 4.6 mm, 5 μm) at 35 *C. mobile* phase: methanol: water (0.1% acetic acid) 65:35 (v/v), flow rate 1.0 mL/min, injection 10 μL, detection at 306 nm. Quantification used external standard calibration of trans‐resveratrol, 0.5–50 μg/mL (Chen et al. 2002).

### 2.3 Animals

Male ICR mice (6 weeks old, weight 27 ± 3) were purchased from Peng Yue Laboratory Co. Ltd., Jinan, China. Four mice were housed per cage under alternating 12‐h light and dark cycles, with room temperature maintained at 25°C ± 2°C and relative humidity at 50% ± 5%. The mice were provided unrestricted access to standard laboratory chow pellets and water ad libitum. The experimental protocol was reviewed and approved by the Animal Ethics Committee of Shandong University of Technology (approval no. SDUTLL2021051001). The care and handling of the mice adhered strictly to the institutional guidelines of Shandong University of Technology.

### 2.4 Treatments and Groups

Following a 1‐week acclimatization period, male ICR mice were randomly assigned to six groups, with each group comprising eight mice. The induction of BPH and the selection of dosages were adapted from established literature with minor modifications to assess the dose‐dependent effects (Jeon et al. 2017). The dosages described below for all the experimental groups were adjusted every third day based on body weight (BW). The experimental groups and dosages were structured as follows: the blank group received daily subcutaneous injections of olive oil and normal saline with oral gavage; negative control (NC) group and all other groups received 6 mg/kg BW/day subcutaneous TP in olive oil to induce BPH. Positive control (PC) group received 1 mg/kg BW oral finasteride. Low‐dose (LD), medium‐dose (MD), and high‐dose (HD) groups received 100, 200, and 300 mg/kg BW/day AHRE, respectively, along with TP. After the last treatment on day 30, mice fasted for 15 h before blood collection. On day 31, mice were euthanized, prostate glands were excised, weighed, and fixed in 10% NFB for histology.

### 2.5 Prostatic Index and Percent Inhibition

On the 31st day, the mice's weight was recorded before euthanizing them by cervical dislocation. Prostate glands were excised and the weight was recorded immediately. To calculate the prostatic index (PI), the following equation was used (Huang et al. 2017):

$$\mathrm{PI}=\frac{\text{Mean prostate weight}}{\text{Mean mouse weight}}$$

While Babu et al. (2010) method was used to calculate the percent inhibition of BPH, the following equation was applied:

$$\text{Percent inhibition}=100-\left(\frac{\text{Treatment}-\text{Blank group}}{\mathrm{NC}\;\text{group}-\text{Blank group}}\times 100\right)$$

### 2.6 Quantification by ELISA

Blood samples of fasted mice (15 h) were collected. The serum was obtained by centrifuging the blood samples at 1500 × *g* for 10 min and stored at −80°C until further analysis (Shanmugasundaram et al. 2024). Samples were quantified for PAP in ng/mL, E_2_ in ng/mL, T in nmol/L, DHT in nmol/L, and AR in ng/mL by using commercially available ELISA kits, details mentioned in Section 2.1.

### 2.7 Histopathological Examination

The paraffin‐embedded prostate glands were sliced to 5 μm thickness. Hematoxylin and eosin (HE) solutions were used to stain the sectioned tissues. The histological analysis was performed according to the reported method of Corica et al. (2000) using 40× magnification to observe the tissue under a light microscope. The histopathological changes were evaluated semi‐quantitatively blindly by two independent observers with the histological scoring system (1–5 scale) (Zhang, Luo, et al. 2020). This system assessed the overall severity of epithelial hyperplasia, stromal proliferation, glandular distortion, and inflammatory infiltration. Scores were averaged across observers and expressed in arbitrary units (AU) as mean ± SD per group. Additionally, the prostatic cells were differentiated based on their morphology as the prostatic epithelial cells, stromal cells, and inflammatory cells and counted in 5 randomly selected high‐power fields (200×) of each slide.

### 2.8 Quantitative Real‐Time PCR

Total RNA was prepared from the collected prostate tissues using an RNA extraction kit (TAKARA 9767 TaKaRa MiniBEST Universal RNA Extraction Kit; code no. 9767). RNAs were reverse transcribed using the cDNA Reverse Transcription Kits (PrimeScriptTM RT reagent Kit with gDNA Eraser; code no. RR047A) according to the manufacturer's instructions. Quantitative real‐time polymerase chain reaction (qRT‐PCR) was performed using the SYBR Green Master Mix (Applied Biosystems) in a Fast 96‐well System (Applied Biosystems). The primer sequences are listed in Table S1. The relative mRNA expression levels of target genes to the loading control *β‐Actin* were calculated with the 2^−ΔΔCT^ method.

### 2.9 16S rRNA Gene Sequencing and Spearman Correlation Analysis of Gut Microbiota With BPH‐Related Phenotypes

For gene sequencing of fecal microbiota, the previously reported method by Ijaz et al. (2020) was adopted and detailed is provided in Supporting Information. Briefly, the TIANamp Stool DNA kit (DP328) was used to extract the genomic DNA from the feces of treated mice. The 515F primer (5′‐barcode‐GTGCCAGCMGCCGCGG‐3′) and the 806R primer (5′‐GGACTACHVGGGTWTCTAAT‐3′) were used to amplify the bacterial ribosomal RNA gene. The amplicons were purified with a DNA gel extraction kit (Axygen Biosciences, Union City, CA, USA) and quantified with a Life Technologies Qubit 3.0 (Carlsbad, CA, USA). The amplicons, individually barcoded, were integrated, and an Illumina library was built using Illumina's genomic DNA library procedures. Paired‐end (2 × 250) sequencing was carried out using an Illumina MiSeq platform (San Diego, CA, USA). QIIME software from qiime2.org was used to process the collected readings. UPARSE software (version 7.1) was used to cluster operational taxonomic units (OTUs) at a 97% similarity threshold, and the RDP classifier was used to classify 16S rRNA genes against the SILVA (SSU123) database. Data were matched with a 70% confidence level (Amato et al. 2013). The Shannon–Weiner diversity index (*H*′), Chao index, and Good's coverage index (Schloss et al. 2009) were calculated to quantify community richness. Clustering analysis and principal coordinate analysis (PCoA) were performed on the OTUs to acquire a better understanding of the microbial composition of the feces (http://sekhon.berkeley.edu/stats/html/hclust.html) (Lozupone et al. 2011). Linear discriminant analysis effect size (LEfSe) analysis was used to differentiate and identify the biological circumstances across different groups and to define biomarkers for colonic bacteria (http://huttenhower.sph.harvard.edu/galaxy/) (Segata et al. 2011).

Spearman correlation coefficients (*ρ*) were calculated between the relative abundance of selected gut microbial genera and key BPH–related phenotypes and markers (including: PW, PI, T and DHT, PAP, AR, E_2_, T/E_2_, *ER‐α*, *COX‐2*, *LOX‐5*, and *HIF‐1α*). These correlations were calculated using treatment‐level mean values across experimental groups and are therefore presented as exploratory, associative analyses rather than evidence of direct causality. *p*‐values were adjusted for multiple comparisons using the Benjamini–Hochberg false discovery rate (BH‐FDR) method. Genera were filtered based on prevalence and variance criteria to reduce sparsity and enhance biological interpretability.

### 2.10 Statistical Analysis

Data from all the experiments are expressed as mean ± SEM. For ELISA and qRT‐PCR data, Tuckey's and Dunnett's multiple comparison tests were performed wherever appropriate followed by one‐way analysis of variance. For gut microbiota, group differences were assessed with *t*‐tests and multivariate analysis of variance, with statistical significance set at *p* < 0.05.

## 3 Results

### 3.1 AHRE Components and Resveratrol

**FIGURE 1:**
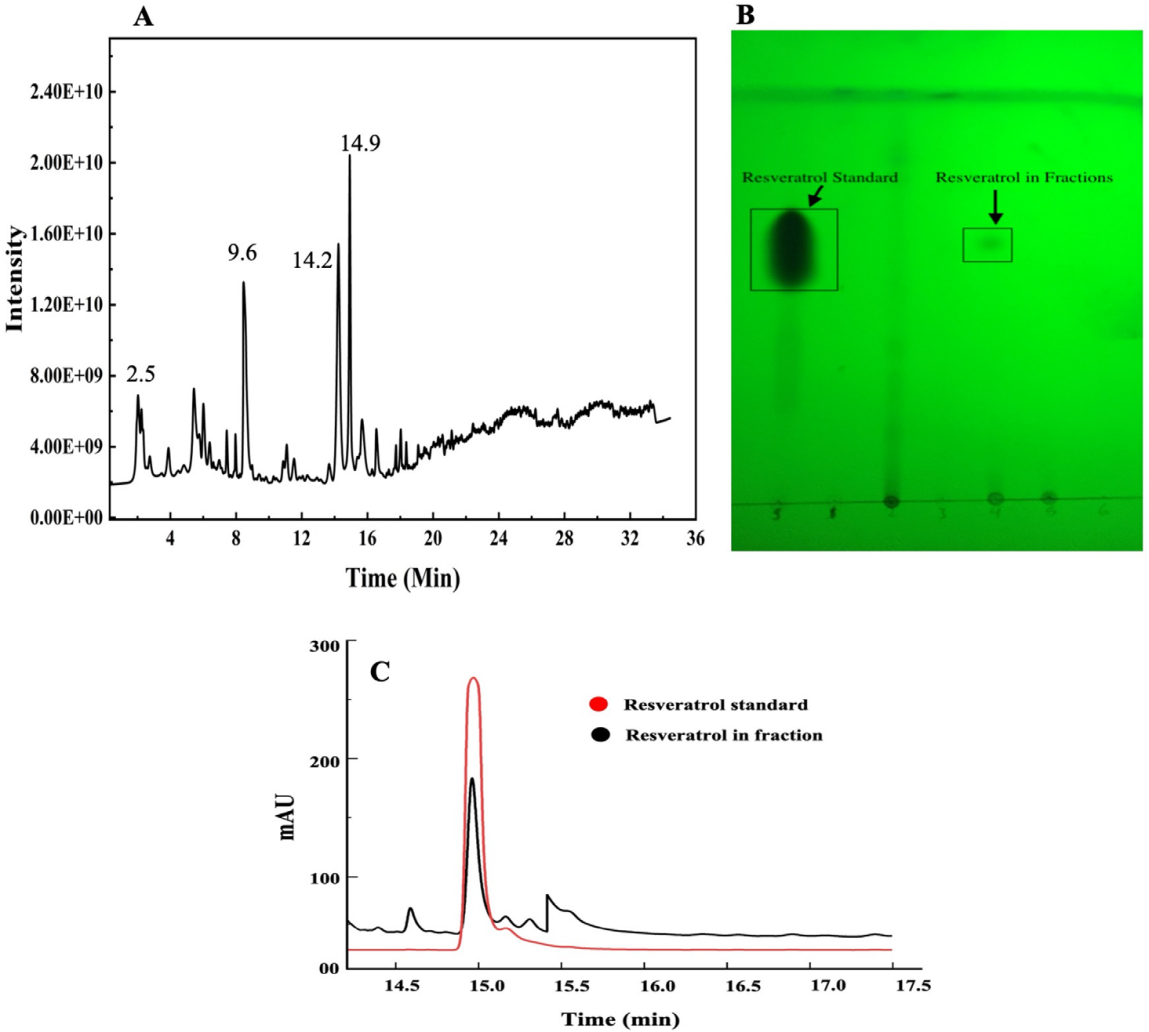
HPLC‐qTOF‐MS chromatogram of AHRE (A), TLC (B), and HPLC analysis of resveratrol containing fraction of AHRE (C).

For HPLC‐QTOF‐MS analysis, peaks corresponding to reference compounds were identified by comparing their mass spectra and retention times with literature data, reference standards, and database entries. A total of 19 compounds (Figure 1A and Table 1) were identified from the AHRE. The TLC‐positive fractions for resveratrol were subsequently collected and analyzed for its concentration using HPLC, which was 1.12 mg/g of extract (Figure 1B,C). This finding is consistent with other studies in the literature, which have reported similar resveratrol concentrations in the AHRE (Hung and Chen 2022).

**TABLE 1:** Identified compounds in 70% ethanolic AHRE.

Peak no.	Compound	RT (min)	Formula	*m/z* [M‐H]^−^	Error (ppm)
1	p‐Coumaric acid	2.5	C_9_H_8_O_3_	163.0401	−0.09
2	Caffeic acid	2.9	C_9_H_8_O_4_	179.0950	−0.95
3	Ferulic acid	3.7	C_10_H_10_O_4_	193.0807	−0.97
4	4‐Methoxycinnamic acid	5.2	C_10_H_10_O_3_	177.0558	−1.0
5	Chlorogenic acid	6.5	C_16_H_18_O_9_	353.0879	−1.90
6	Piceatannol	9.6	C_14_H_12_O_4_	245.9667	1.89
7	Daidzein	11.3	C_15_H_10_O_4_	254.8526	0.79
8	Cis‐Resveratrol	14.2	C14H12O3	227.7018	0.38
9	Trans‐Resveratrol	14.9	C_14_H_12_O_3_	227.9732	0.11
10	Genistein	16.5	C_15_H_10_O_5_	269.0456	−0.4
11	Formononetin	17.3	C_16_H_12_O_4_	267.2063	−0.86
12	Luteolin	18.8	C_15_H_10_O_6_	285.0405	−0.93
13	Demethylmedicarpin	19.6	C_15_H_12_O_4_	258.1663	2.9
14	Medicarpin	21.4	C_16_H_14_O_4_	272.0820	1.99
15	t‐3′‐Isopentadienyl‐3,5,4′‐trihydroxystilbene	23.7	C_19_H_20_O_3_	293.8517	−2.29
16	Arachidin‐1	27.5	C_20_H_22_O_3_	311.1293	0.98
17	Arachidin‐3	29.7	C_19_H_20_O_3_	298.2592	2.11
18	Arachidin‐2	30.1	C_20_H_22_O_4_	328.1242	1.97
19	Mucilagin	31.3	C_21_H_24_O_3_	327.2139	3.04

### 3.2 Mice BW, PW, PI, Prostate Tissue Histology and Prostatic Cell Count

The first step in evaluating the impact of the treatments at the study's conclusion involved measuring BW of animals. No notable differences were observed in the BW values among the experimental groups. Although the NC group has shown a significant increase (*p* < 0.0001) in PW 64.399 ± 3.866 mg (a 68.62% increase) and PI (66.91% increase) as compared to the blank group (PW: 38.192 ± 1.367 mg). The AHRE HD group showed the maximum inhibition with *p* < 0.0001 (PW and PI inhibited by 84.073% and 33.01%, respectively), while the AHRE MD group showed the second highest percent inhibition i.e., 69.672%, and 27% for PW and PI, respectively (Table 2). Moreover, the total histoscore was highest in the NC group (64.16 ± 5.26). The AHRE LD group did not differ significantly from NC group (58.05 ± 4.11 AU; *p* = 0.0813). AHRE MD group (42.30 ± 5.16) and AHRE HD group (30.01 ± 3.67) produced progressive and significant reductions compared to NC group (*p* < 0.0001 for both) and to lower dose (*p* < 0.0001). The PC group (25.38 ± 2.81) showed the lowest total histoscore, comparable to AHRE HD group (*p* = 0.2811). These results (Table 2) indicate a clear dose‐dependent protective effect of AHRE on BPH‐associated histopathological changes.

**TABLE 2:** Effect of AHRE on mice BW, PW, prostate size inhibition, PI, and total histoscore.

Treatment groups	BW (g)		PW (mg)	Prostate size inhibition (%)	PI (10^−3^)	Total histoscore (AU)
	0 Day	31st day				
Blank	35.41 ± 1.409^a^	35.96 ± 1.638^a^	38.192 ± 1.367^e^	—	1.065 ± 0.071^d^	—
NC	34.263 ± 1.7^a^	36.354 ± 2.087^a^	64.399 ± 3.866^a^	—	1.778 ± 0.148^a^	64.16 ± 5.26^a^
PC	35.784 ± 1.889^a^	36.498 ± 1.902^a^	40.023 ± 1.403^de^	93.103^a^	1.1 ± 0.082^d^	25.38 ± 2.81^c^
AHRE LD	34.001 ± 1.825^a^	35.841 ± 2.176^a^	51.339 ± 1.814^b^	48.699^d^	1.479 ± 0.098^b^	58.05 ± 4.11^a^
AHRE MD	34.613 ± 1.424^a^	35.502 ± 1.899^a^	45.984 ± 1.168^c^	69.672^c^	1.298 ± 0.073^c^	42.30 ± 5.16^b^
AHRE HD	35.813 ± 2.052^a^	36.572 ± 1.959^a^	42.137 ± 2.516^d^	84.073^b^	1.191 ± 0.127^cd^	30.01 ± 3.67^c^

*Note:* Within each column, the different superscripted letters indicate statistical significance (*p* < 0.05).

**FIGURE 2:**
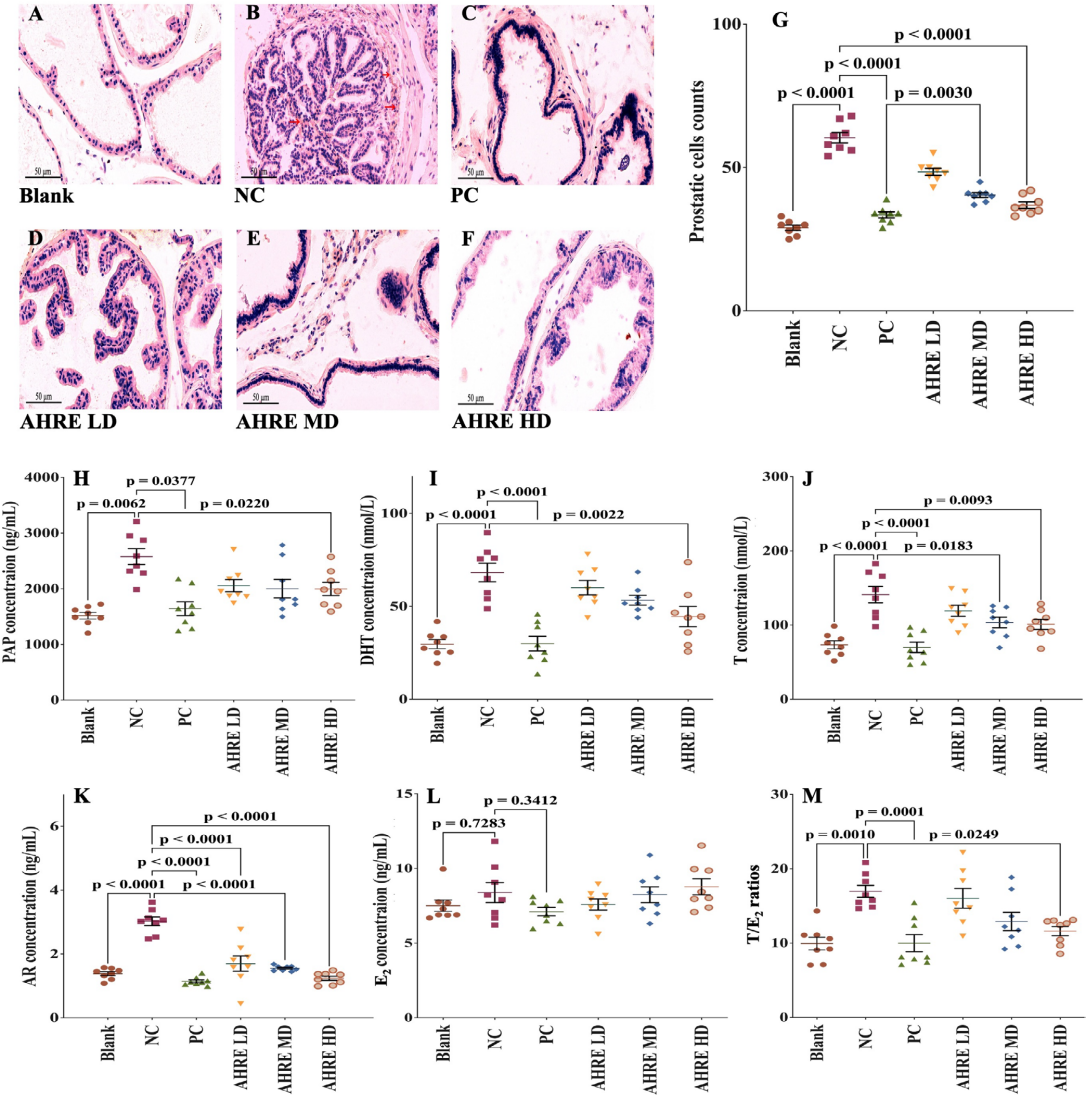
Effect of AHRE on the histopathology (A–F), prostatic cell count (G), PAP (H), DHT (I), T (J), AR (K), T/E_2_ (L), and T/E_2_ ratios (M) of mice. AHRE, *Arachis hypogaea* L. root extract; HD, High dose; LD, Low dose; MD, Medium dose; NC, Negative control; PC, Positive control; *n* = 8.

The histopathological examination of the prostate tissue is one of the irreplaceable methods for diagnosis. The protective effect of AHRE along with the control groups has been depicted in Figure 2A–F. Moreover, compared to the blank group, glandular hyperplasia of the prostate tissue, characterized by muscle thickening due to multiple layers of epithelial cells, is clearly evident in the NC group (Figure 2A,B). In the finasteride treated PC group, the formation of glandular hyperplasia as well as prostatic cell count (Figure 2C,G) were reduced in comparison to the NC.

Whereas, among the treated groups, the AHRE HD (Figure 2F) group had a comparable effect on glandular hyperplasia to that of the PC group. Additionally, prostatic cell count analysis revealed an approximate 80% increase in the NC group compared to the blank group. Notably, all treatment groups exhibited a significant (*p* < 0.05) reduction, with prostatic cell counts decreasing from 60.4 ± 4.8 in the NC group to 33.5 ± 2.8 in the finasteride PC group and 36.8 ± 3 in the AHRE HD group (Figure 2G). Furthermore, both prostatic cell count and histological examination indicated that the AHRE LD treated group did not significantly reduce the formation of glandular hyperplasia.

### 3.3 Mice Serum T, DHT, PAP, AR, E_2_ , and T/E_2_ Ratios

Furthermore, in this study, serum PAP concentration levels were used to evaluate the relationship between BPH activity and AHRE. The analysis of the serum PAP indicates a significantly (*p* < 0.0377) higher (2377.261 ± 187.589 ng/mL) in the NC group as compared to the PC group (1608.111 ± 116.495 ng/mL) and to the blank group (1554.896 ± 181.542). The treatment groups showed significantly (*p* < 0.05) lower serum levels of PAP as compared to the NC group (Figure 2H). The best results (*p* < 0.022) were produced by the AHRE HD group (1597.098 ± 88.249 ng/mL) among the AHRE treated groups.

Moreover, serum T and DHT levels in the NC group were significantly (*p* < 0.0001) elevated as compared to the blank group. Finasteride PC group had the lowest T and DHT serum levels (Figure 2I,J). Among the AHRE treated groups, the lowest level of DHT (31.357 ± 15.19 nmol/L) was observed in the AHRE HD group which was significantly (*p* < 0.0022) lower than the NC group. It was also noted that the differences in the mean serum T levels among the AHRE treated groups were not significant (Figure 2J). Moreover, due to the higher DHT concentration in the NC group, AR concentrations were significantly higher (*p* < 0.0001) compared to the blank group (Figure 2K). The blank group and finasteride PC groups exhibited no significant difference in the AR levels. Notably, all AHRE‐treated groups demonstrated inhibition of AR. Furthermore, no significant difference was recorded in the E_2_ levels among the AHRE treatment groups (Figure 2L). However, a significant (*p* < 0.0001) difference in T/E_2_ ratio was observed in the NC group due to the increased level of T as compared to the blank group and the PC group. The T/E_2_ ratio among the AHRE treated groups was reduced significantly (*p* < 0.0249) in the AHRE HD group compared to the NC group, as depicted in Figure 2M.

### 3.4 AHRE Mediated the Hypoxia Inducible Factor (*HIF‐1α*) and Inflammation in the Mice's Prostate

**FIGURE 3:**
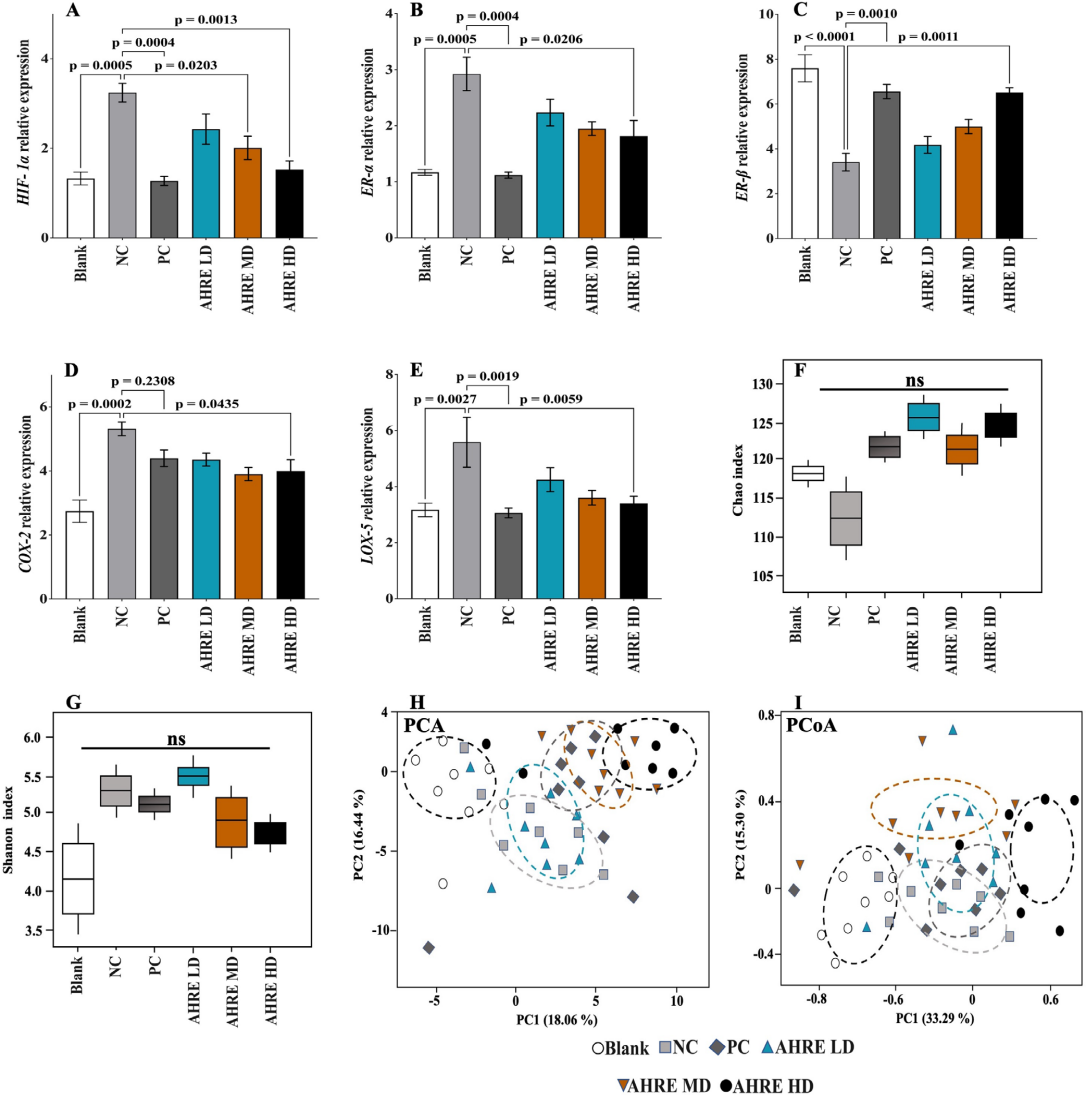
Effect of AHRE on the relative expression of *HIF‐1α* (A), *ER‐α* (B), *ER‐β* (C), *COX‐2* (D), *LOX‐5* (E), Chao index (F), Shannon index (G), PCA (H), and PCoA (I). AHRE, *Arachis hypogaea* L. root extract; HD, high dose; LD, low dose; MD, medium dose; NC, negative control; PC, positive control, *n* = 8.

The relative expression of the *HIF‐1α* increased significantly (*p* = 0.0005) in the NC group compared to the blank group (Figure 3A). Among the other AHRE‐treated groups, the change in *HIF‐1α* relative expression was non‐significantly compared to the blank group. The maximum inhibition (*p* = 0.0004) was shown by the finasteride PC group, which is already established among the first‐line treatments for the management of BPH. Recent studies have demonstrated that *HIF‐1α* is highly expressed in BPH, probably because an enlarged prostate gland and compromised blood flow within the enlarged tissue lead to increased *HIF‐1α* relative expression as a compensatory mechanism (Chen, Xu, et al. 2019). Furthermore, the impact of AHRE on the relative expressions of *estrogen receptor alpha* (*ER‐α*), *estrogen receptor beta* (*ER‐β*), *lipoxygenase‐5* (*LOX‐5*), and *cyclooxygenase‐2* (*COX‐2*) in the prostate tissues of mice is depicted in Figure 3A–E. Treatment with AHRE HD resulted in a significant downregulation of *ER‐α* (*p* = 0.0206) and *ER‐β* expression was raised (*p* = 0.0011) (Figure 3B,C). The raised level of *ER‐α* in the NC group indicated increased activity of aromatase, which promotes inflammation. The *LOX‐5* relative expression was increased (≅2 folds) in the NC group (*p* = 0.0027) compared to the blank group. Among the treatment groups, all groups showed a reduction in *COX‐2* and *LOX‐5* relative expressions; however, after the finasteride PC group, marked reduction was produced by AHRE HD with *p* = 0.0435 and *p* = 0.0059 for *COX‐2* and *LOX‐5* relative expressions, respectively. These reductions were comparable to those observed in the finasteride PC group (Figure 3D,E).

### 3.5 Mice Gut Microbiota Diversity

A total read count of 121,642 ± 7634 from the blank group, 118,364 ± 6439 from NC group, 134,456 ± 7469 from PC group, 129,934 ± 5961 from AHRE LD group, 135,028 ± 5547 from AHRE MD group, and 139,364 ± 5748 from the AHRE HD group were recorded. The number of reads in the sequencing data and the number of OTUs did not exhibit a statistically significant difference between the groups (*p* > 0.05). Furthermore, with 25,000 qualified sequences, it is evident that the sequencing depth and quantity fulfilled the requirements for both sequencing and analysis, effectively encompassing a broad spectrum of diversity. Additionally, the OTU rank abundance curve exhibited a wide, gradual decline, indicating a high level of abundance and even distribution (Figure S1). Moreover, no significant influence on measures of community richness (Chao and good's coverage) and diversity (Shannon and Simpson indices) among the treated groups was recorded. However, in NC group, lower chao and good's coverage values were recorded compared to the other AHRE treated groups and the blank group (*p* < 0.05, Figure 3F,G).

### 3.6 Microbiota Composition and Functionality

The findings of principal component analysis (PCA) revealed that the makeup of the microbiota varied among the different treatment groups, regardless of whether the mice were given AHRE in LD or HD (Figure 3H). The first two major components explained 34.50% of the entire variance in gut microbiota composition. Pairwise PERMANOVA analysis further confirmed the strongest effects in pairs involving medium and high doses of AHRE extract versus control. Pseudo‐*F* values were markedly elevated in dose‐contrasting comparisons (e.g., NC group vs. AHRE HD group: pseudo‐*F* = 79.06, *p* = 0.007, *q* = 0.009; AHRE LD group vs. AHRE HD group: pseudo‐*F* = 77.91, *p* = 0.007), indicating pronounced multivariate dissimilarity with increasing dose. Non‐significance was observed between NC group vs. AHRE LD group (pseudo‐*F* = 3.10, *p* = 0.068, *q* = 0.068). The PC group differed significantly from most groups but showed moderate pseudo‐*F* values.

**FIGURE 4:**
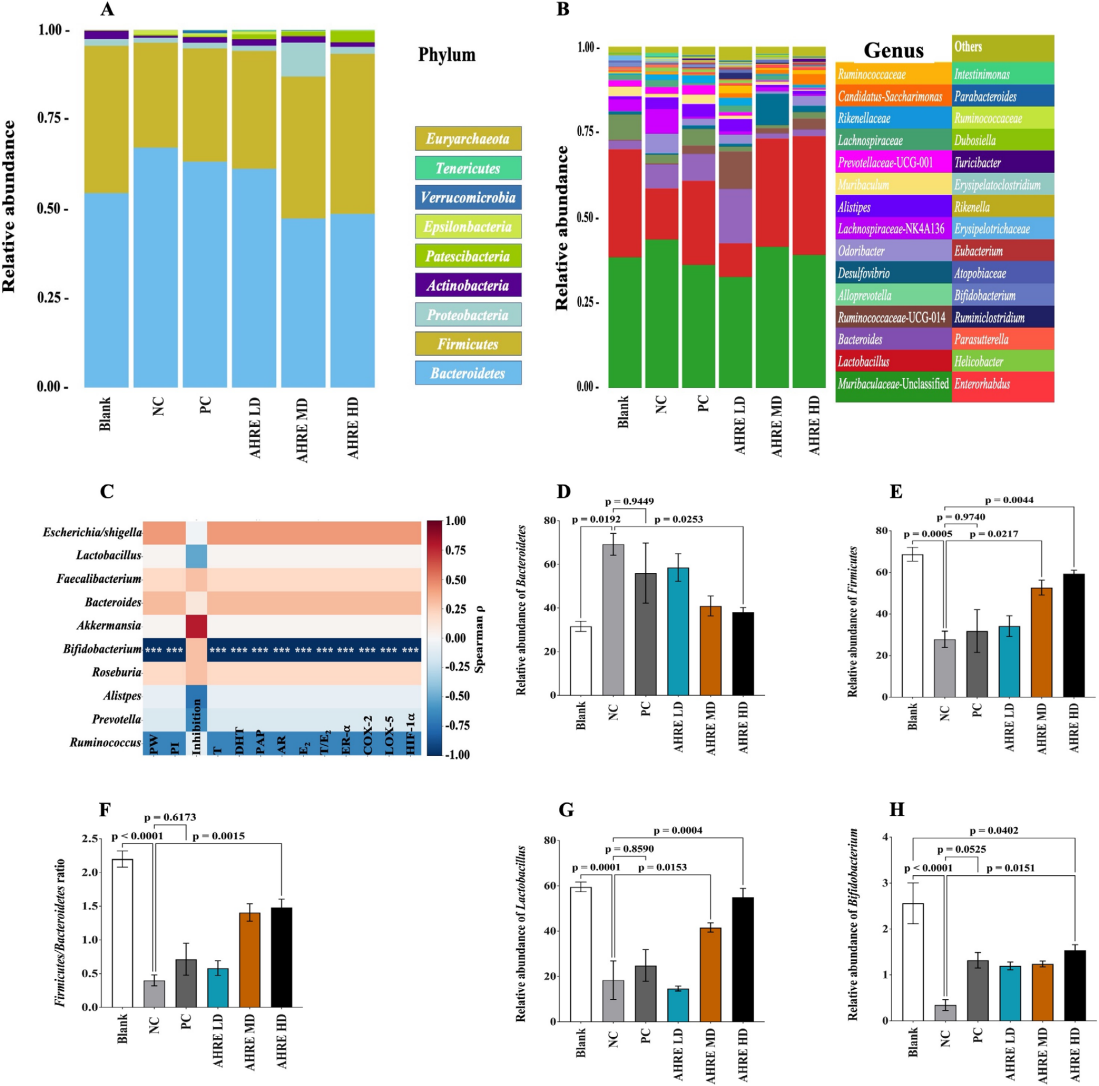
Relative abundance at phylum (A), genus levels (B), genus‐phenotype correlation heatmap (BH‐FDR corrected) * (C), relative abundance of *Bacteroidetes* (D), *Firmicutes* (E), *Firmicutes/Bacteroidetes* ratios (F) at phylum levels, *Lactobacillus* (G), and *Bifidobacterium* (H) at genus levels. AHRE, *Arachis hypogaea* L. root extract; HD, high dose; LD, low dose; MD, medium dose; NC, negative control; PC, positive control, *n* = 8. *Color intensity reflects the direction and strength of association (red: Positive; blue: Negative).

Pairwise PERMANOVA analysis of principal coordinates analysis (PCoA) was employed to assess these differences and revealed a pattern of group separations consistent with the observed ordination. While fewer comparisons reached strong significance compared to PCA, dose‐dependent contrasts involving higher AHRE doses remained highly significant with elevated pseudo‐*F* values (e.g., Blank vs. AHRE HD: pseudo‐*F* = 34.02, *p* = 0.007, *q* = 0.015; NC group vs. AHRE HD group: pseudo‐*F* = 46.86, *p* = 0.007; PC group vs. AHRE HD group: pseudo‐*F* = 27.49, *p* = 0.007) as depicted in Figure 3I. At the phylum level, the dominant bacterial groups were *Bacteroidetes* and *Firmicutes*. Although there were no statistically significant distinctions for *Bacteroidetes* and *Firmicutes* (*p* > 0.05). It's worth noting that *Firmicutes* exhibited a decreasing tendency, while *Bacteroidetes* displayed an increasing trend when the NC group was compared to the blank group, AHRE MD, and AHRE HD groups (Figure 4A).

### 3.7 AHRE Modulates Gut Microbiota to Prevent BPH

By analyzing the taxonomic distribution, significant distinctions in the flora at the genus, family, and phylum levels were observed when comparing the PC group with the blank and AHRE treated groups, particularly the AHRE MD and HD groups (Figure 4). At the phylum level, these AHRE treated groups significantly (*p* < 0.05) reduced the abundance of *Bacteroidetes* (*B*) compared to the NC group, accompanied by a significant increase in the abundance of *Firmicutes* (*F*) and an improved ratio of *F/B* (Figure 4B–E). Furthermore, in comparison to the blank group, AHRE treated groups exhibited reduced *Bacilli* as well as increased levels of *Bacteroidales* and *Clostridia*. At the genus level, the relative abundance of Ruminococcaceae, *Lactobacillus*, and *Bifidobacterium* increased significantly (*p* < 0.05) in the AHRE MD and HD groups as compared to the NC and PC groups, as depicted in Figure 4G,H. Moreover, relative abundance of *Lactobacillus* at the genus level was also observed to be significantly (*p* < 0.0001) reduced in the PC group as compared to the blank group (Figure 4F). Similarly, the LEfSe analysis discovered significant differences in OTUs across the 6 mice groups for the taxonomic distribution of certain microbial species, from phylum to genus.

### 3.8 Integrative Correlation of Gut Microbial Genera With BPH Phenotypes

To explore coordinated changes in the gut microbiota and host responses following AHRE treatment, we performed Spearman correlation analyses between treatment‐level mean relative abundances of prevalent, high‐variance bacterial genera and a panel of BPH‐related phenotypes (Figure 4C). The selected phenotypes included PW, PI, prostate size inhibition rate, circulating T and DHT, PAP, AR, E_2_, T/E_2_, *ER‐α*, and inflammatory/hypoxia markers *COX‐2*, *LOX‐5*, and *HIF‐1α*.

*Bifidobacterium* abundance was robustly and negatively correlated with PW (*ρ* = −0.95, *q* < 0.001), PI (*ρ* = −0.96, *q* < 0.001), and multiple inflammatory markers (*COX‐2*, *LOX‐5*, *HIF‐1α*; all *q* < 0.001), consistent with a potential protective role against prostate enlargement and inflammation. Conversely, *Akkermansia* abundance showed a strong positive correlation with inhibition rate (*ρ* = 0.87, *q* < 0.01), aligned with its reported anti‐inflammatory and barrier‐enhancing functions. *Ruminococcus* and *Alistipes* were negatively associated with DHT and PAP (*ρ* range: −0.68 to −0.72, *q* < 0.05), suggesting modulation of androgen metabolism pathways. *Lactobacillus* exhibited a moderate negative association with inhibition (*ρ* = −0.53, *q* < 0.05) but did not correlate significantly with inflammatory markers. Other genera, such as *Faecalibacterium* and *Roseburia*, demonstrated weak positive associations with T/E_2_ (*ρ* ~ 0.30, *q* > 0.05), indicating possible links to estrogenic balance.

These exploratory, treatment‐level associations highlight a coordinated gut–prostate axis, in which shifts in specific microbial taxa parallel improvements in prostate hypertrophy, androgenic hormone balance, and inflammatory status following AHRE intervention.

## 4 Discussion

BPH is a multifactorial condition driven by complex molecular mechanisms, including hormonal dysregulation, chronic inflammation, oxidative stress, and as emerging evidence suggests, gut microbiota imbalances. Diagnostic tests like prostate phosphate and PAP are often used for the detection of BPH and prostate cancer (Graddis et al. 2011). The released phosphate from the prostate can be hydrolyzed by the enzyme acid phosphatase and ultimately used as a biochemical marker to determine the progress of BPH development. This makes it imperative in the regulation of prostate growth and cell proliferation in the prostate gland (Eri and Tveter 2001). The pathogenesis of BPH is linked to androgenic stimulation of prostatic tissues, primarily mediated through the conversion of T to DHT by 5*α*‐reductase isoenzymes. DHT binds with higher affinity to the AR and stimulates the transcription of genes that promote cellular proliferation and inhibit apoptosis in prostate epithelial and stromal cells (Chen, Chen, et al. 2019). Moreover, the synthesis of the T in the testis started to boost after the activation of the AR. The presence of resveratrol in AHRE inhibited the formation of DHT formation (Zhang et al. 2021) and it also exhibits anti‐proliferative and pro‐apoptotic effects in human BPH‐1 cells (Li et al. 2019; Meng et al. 2021). Thus, AHRE may therefore provide both cellular and symptomatic relief by targeting both epithelial and stromal cells. Despite the moderately increased T levels in AHRE‐treated groups, the overall androgenic stimulation of the prostate was reduced, possibly due to the reduced conversion of T to DHT and the ultimately lowered AR activity. Moreover, AHRE normalized the T/E_2_ ratio and restored hormonal homeostasis, as an unfavorable T/E_2_ ratio is associated with enhanced aromatase activity and increased stromal cell proliferation.

AHRE contributed to the stabilization of *HIF‐1α* relative expression, likely through its anti‐inflammatory, anti‐proliferative, and antioxidant properties. Previous studies have documented the role of resveratrol as a *HIF‐1α* modulator by inhibiting PI3K/Akt/mTOR signaling and thereby limiting hypoxia signaling in several tissue types (He et al. 2025; Hedayati et al. 2025). Thus, AHRE likely attenuated the cascade of hypoxia and oxidative stress by reducing vascular congestion and cellular overgrowth, thereby preventing *HIF‐1α* activation. Although androgens are the primary drivers of prostatic growth, estrogens also exert profound effects through their receptors *ER‐α* and *ER‐β*. The significant reduction in glandular hyperplasia observed in the AHRE HD group may thus be partially attributed to the modulation of *ER‐α* and *ER‐β* signaling. This is because *ER‐α* is generally associated with inflammatory, proliferative, and pro‐fibrotic effects, whereas *ER‐β* opposes these actions by promoting anti‐proliferative and apoptotic pathways (Dey et al. 2014). Furthermore, chronic inflammatory cell infiltration leads to increased expression of pro‐inflammatory mediators such as *LOX‐5* and *COX‐2* (Cohen et al. 2006). They facilitate the biosynthesis of leukotrienes and prostaglandins, respectively—key mediators in the perpetuation of inflammation and resistance to apoptosis. Their upregulation also enhances the expression of anti‐apoptotic proteins such as Bcl‐2, sustaining cell survival (Haeggstrom and Newcomer 2023; Jin et al. 2019). The downregulation of *LOX‐5* and *COX‐2* observed following AHRE treatment strongly indicates suppression of inflammatory signaling.

The stronger separations in PCA likely reflect its emphasis on maximum variance, while PCoA's distance‐based nature better captures overall dissimilarity patterns but with reduced sensitivity at lower effect sizes (Figure 3H,I). Our integrative correlation analysis (Figure 4C) provides compelling exploratory evidence that gut microbial composition shifts in concert with key BPH outcomes under AHRE treatment. The strong negative correlations between *Bifidobacterium* abundance and both prostate enlargement (PW, PI) and inflammatory markers (*COX‐2*, *LOX‐5*, *HIF‐1α*) are particularly noteworthy. *Bifidobacterium* species are known producers of short‐chain fatty acids (SCFAs), especially acetate, which exert anti‐inflammatory effects and enhance mucosal integrity; their enrichment may therefore contribute to systemic modulation of inflammation and downregulation of prostatic *COX/LOX* pathways (Parada Venegas et al. 2019). *Akkermansia*'s positive association with inhibition rate aligns with its well‐characterized role in maintaining epithelial barrier function and attenuating metabolic inflammation. The negative correlations observed for *Ruminococcus* and *Alistipes* against DHT and PAP further suggest that microbiota may influence host steroid metabolism, possibly via microbial β‐glucuronidase activity or bile acid signaling that impacts 5α reductase and AR pathways. Although *Lactobacillus* did not show significant associations with inflammatory markers, its moderate correlation with inhibition rate may reflect strain‐specific effects on androgen metabolism. *Faecalibacterium* and *Roseburia* exhibited modest links to T/E_2_ ratio, hinting at potential microbial contributions to estrogenic modulation; however, these associations did not reach statistical significance after BH‐FDR correction and thus warrant further validation.

**FIGURE 5:**
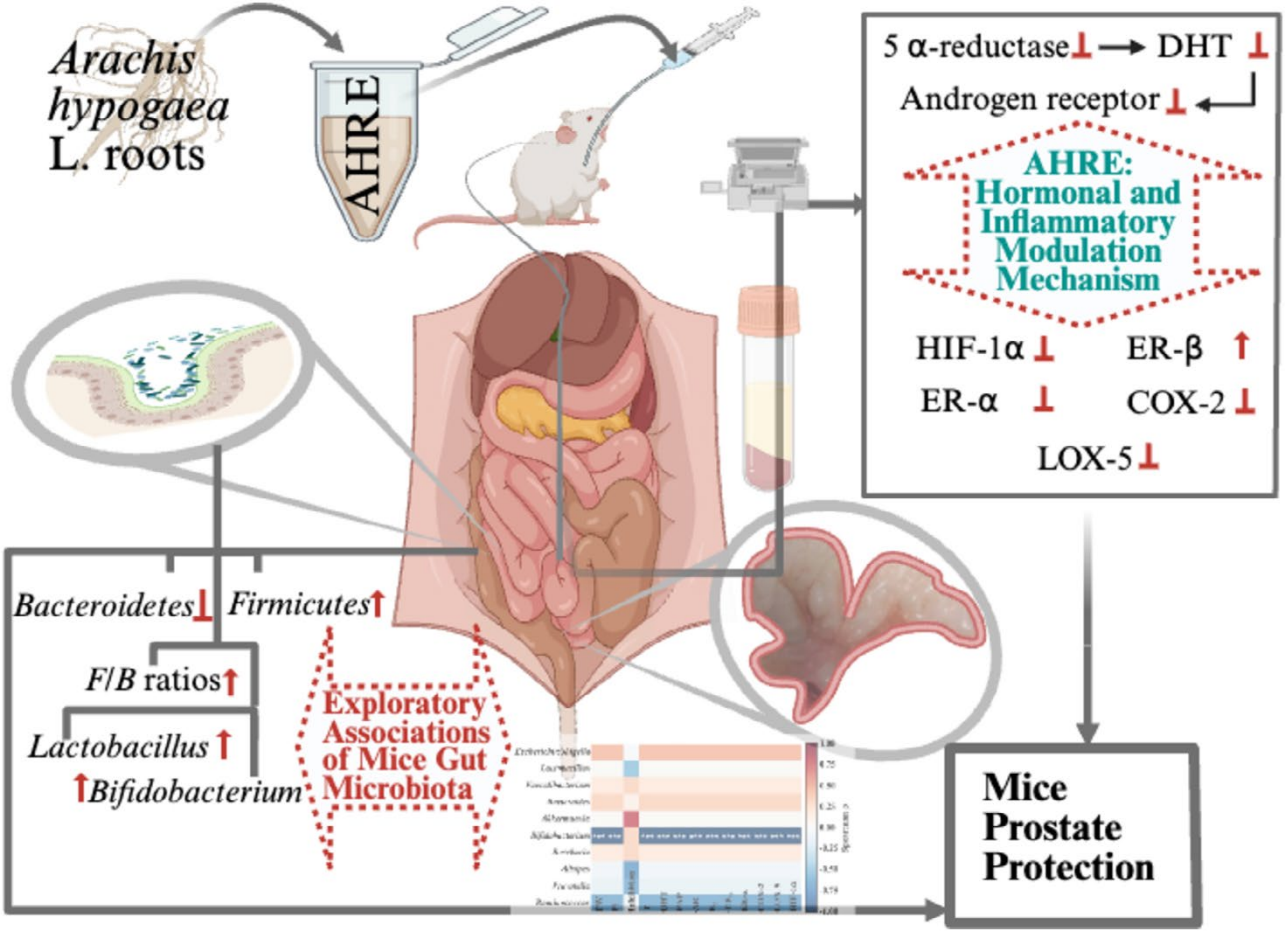
Proposed hormonal and anti‐inflammatory mechanism of AHRE and exploratory associations of mouse gut microbiota with prostate protection.

Moreover, the *F/B* ratio is a well‐established metric for evaluating gut microbiota balance and has been associated with a variety of health conditions. Depression has been shown to induce significant reductions in *Firmicutes* and has been implicated in the development of BPH and LUTS (Kim et al. 2016; Vela‐Navarrete et al. 2018). Analysis of prostate biopsies from BPH patients revealed *F/B* ratio alterations that mirrored those found in the gut microbiota of mice in the present study (Takezawa et al. 2021). The gut microbiome also influences host metabolism by producing bioactive metabolites and participating in hormone regulation, including enterohepatic recirculation of T and conversion of glucocorticoids into androgens (Cross et al. 2018; Pascale et al. 2018). For instance, *Escherichia/Shigella* strains have been shown to increase T levels in adult male mice (Zhang et al. 2020). Our findings demonstrate that AHRE treatment preserves *Bifidobacterium* and *Lactobacillus*. These genera are recognized producers of SCFAs—key metabolites that suppress inflammation, enhance gut barrier integrity, and modulate immune and hormonal pathways. Overall, Figure 4C underscores the presence of a dynamic gut–prostate axis in this BPH model and identifies candidate microbial targets—*Bifidobacterium*, *Akkermansia*, *Ruminococcus*, and *Alistipes*—for future interventional studies aimed at harnessing the gut microbiota to ameliorate prostate hypertrophy and inflammation. Clinical data also link elevated branched‐chain fatty acids to BPH (Ratajczak et al. 2021), which may reflect dysregulated gut fermentation. SCFAs can further influence prostate growth via IGF‐1 and MAPK/PI3K signaling and exert potent immunomodulatory effects by regulating IL‐6, IL‐18, Treg/Th17 balance, and macrophage polarization (Zha et al. 2023). Furthermore, a reduced abundance of beneficial genera such as *Lactobacillus* and *Bifidobacterium* has also been linked to an increased risk of inflammatory diseases, including BPH (Million et al. 2012; Zhang et al. 2022). Hence, the microbiota's ability to modulate androgen metabolism, immune function, and mucosal barrier maintenance may further contributed to the protective effect of AHRE. Figure 5 illustrates the proposed protective hormonal and anti‐inflammatory mechanism of AHRE, along with the exploratory associations between gut microbiota shifts and BPH outcomes.

Furthermore, AHRE demonstrated no adverse effects on body weight and organs in mice. Combined with its dose‐dependent efficacy, this proved AHRE to be a promising phytotherapeutic alternative. Since current BPH pharmacotherapies, such as finasteride and phosphodiesterase inhibitors, while effective, are associated with side effects in long‐term usage. Alternative and effective phytotherapeutics can be attractive choices to treat and ease the symptoms of BPH. Despite these findings, the study is limited by the absence of direct metabolomic profiling and detailed analyses of proliferation/apoptosis pathways. Gut microbiota data were analyzed at the group level and presented as exploratory associations without causal evidence. Future work should validate AHRE's mechanisms through molecular and protein‐level assessment of proliferation and apoptosis pathways, individual‐level microbiome‐metabolome integration (including SCFA and bile acid quantification), and shotgun metagenomics for improved functional inference.

## 5 Conclusion

In summary, the therapeutic potential of AHRE likely stems from the synergistic effects of resveratrol and other bioactive components, which inhibit the formation of glandular hyperplasia in the prostate by inhibiting DHT formation, inflammation, and AR levels, and by protecting against changes in sex hormones. Moreover, AHRE downregulates the expression of *ER‐β*, *LOX‐5*, and *COX‐2* and also protects against changes in the relative abundance of beneficial gut microbiota, which are important for gut lining and reducing systemic inflammation. The current findings are promising and support the traditional use of AHRE to enhance the QoL of BPH patients. However, large‐scale human clinical trials are required to confirm the long‐term efficacy and safety of AHRE.

## Author Contributions

S.U. have major contributions in conceptualization, data curation, formal analysis, funding acquisition, investigation, methodology, and writing – original draft. F.S. have major contributions in conceptualization, formal analysis, investigation, methodology, and writing – original draft. M.U.I. contributed in methodology, software, validation, and writing – review and editing. M.B. contributed in software, writing – review and editing. A.A.K. contributed in methodology, writing – review and editing. T.A. contributed in animal handling, writing – review editing. Y.G. contributed in writing – review editing and visualization. X.S. and Y.S. contributed in conceptualization, methodology, project administration, supervision, validation, visualization, writing – review and editing. M.M.Q. contributed in writing – review editing and visualization.

## Funding

This work was supported by Shandong University of Technology Research Startup Fund Under China Postdoctoral Science Foundation (no. 524043/4041).

## Conflicts of Interest

The authors declare no conflicts of interest.

## Supporting information


**Table S1:** Primer sequences for qRT‐PCR.
**Figure S1:** OTU rank abundance curve.

## Data Availability

The data that support the findings of this study are available from the corresponding authors upon reasonable request.
