# Supplementary material for: Arachis hypogaea L. Root Extract Mitigates Testosterone Propionate‐Induced Benign Prostatic Hyperplasia in ICR Mice by Suppressing Inflammation, Androgen Receptors, and Dihydrotestosterone
Source: Food Sci Nutr. 2026 Mar 23;14(3):e71682. doi: 10.1002/fsn3.71682 (PMC13093758; doi:10.1002/fsn3.71682)
Supplement: Supplementary file 1 — Table S1: Primer sequences for qRT‐PCR. Figure S1: OTU rank abundance curve. [file FSN3-14-e71682-s001.docx]

**Title: *Arachis hypogaea* L. root extract mitigates testosterone propionate-induced benign prostatic hyperplasia in ICR mice by suppressing inflammation, androgen receptors, and dihydrotestosterone**

**Samee-Ullah^1,2,3 #^, Faryal Shaukat^1,2 #^, Muhammad Umair Ijaz^4^, Muhammad Bilal^1,2^, Anees Ahmed Khalil^3^, Tausif Ahmad^1,2^, Yemin Guo^1,2^, Xia Sun^1,2^ *, Yuanda Song^1,5,6^ *, Mohammed Mansour Quradha^7,8^***

**Supplementary Table 1. Primer sequences for qRT-PCR**

| **Genes** | **Primer sequence** | |
| --- | --- | --- |
|  | Forward Primer | Reverse Primer |
| *ER-α* | GGACCATATCCACCGAGTCCTG | GCCTCCCCCGTGATGTAATAC |
| *ER-β* | AGAGTCCCTGGTGTGAAGCAA | GACAGCGCAGAAGTGAGCATC |
| *HIF-1α* | CTATGGAGGCCAGAAGAGGGTAT | CCCACATCAGGTGGCTCATAA |
| *COX-2* | AGAGCAGAGAGATGAAATACC | AGGAGAACAGATGGGATTAC |
| *LOX-5* | TTACGTTTATGGCATGCGGG | GCATAGTTGGAGGAGCGTTG |
| *β-Actin* | TGACAGGATGCAGAAGGAGA | GCTGGAAGGTGGACAGTGAG |

**Supplementary Figure 1. OTU rank abundance curve**

| **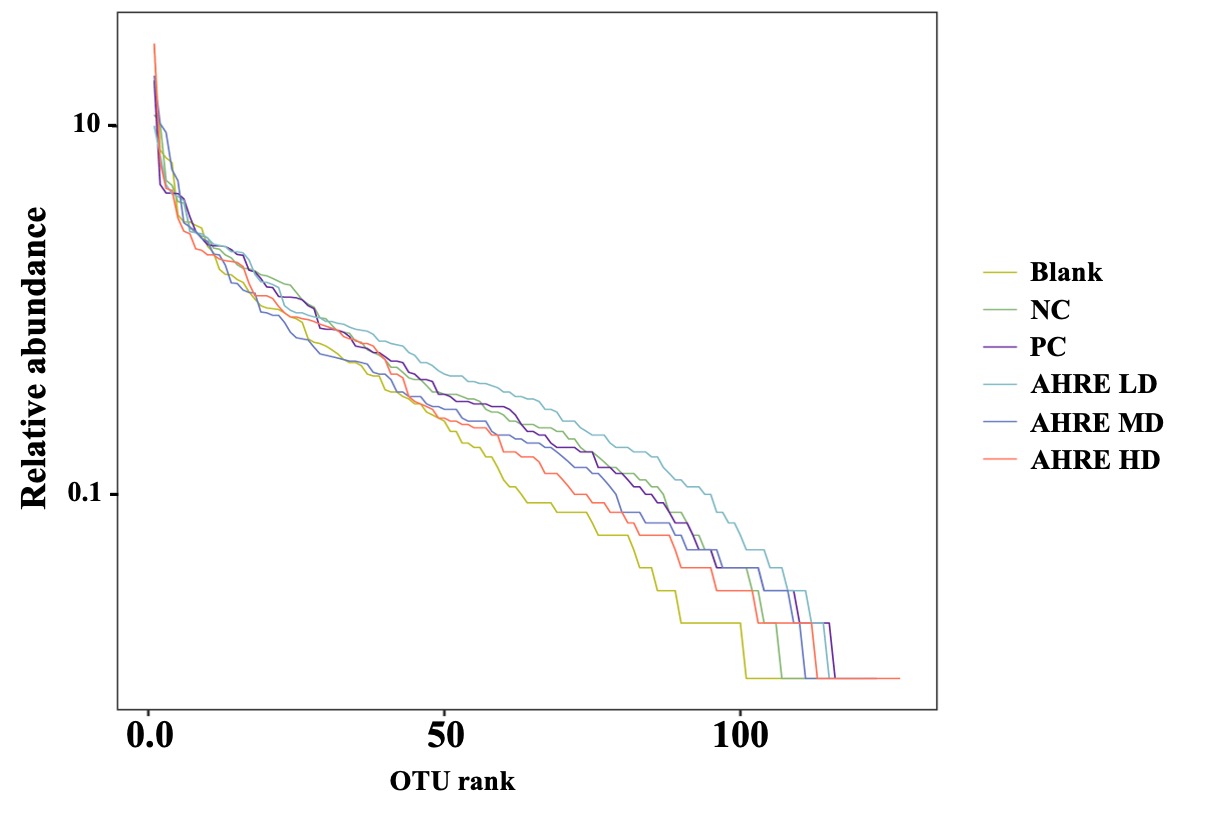** |
| --- |
